# Preclinical and clinical trials of oncolytic vaccinia virus in cancer immunotherapy: a comprehensive review

**DOI:** 10.20892/j.issn.2095-3941.2023.0202

**Published:** 2023-08-23

**Authors:** Mengyuan Li, Minghuan Zhang, Qian Ye, Yunhua Liu, Wenbin Qian

**Affiliations:** 1 Department of Hematology, the Second Affiliated Hospital, Zhejiang University School of Medicine, Hangzhou 310009, China; 2 Hangzhou Rong-Gu Biotechnology Limited Company, Hangzhou 310056, China; 3 Department of Pathology & Pathophysiology and Department of Surgical Oncology of the Second Affiliated Hospital, Zhejiang University School of Medicine, Hangzhou 310058, China

**Keywords:** Oncolytic virotherapy, oncolytic vaccinia virus, engineered virus, arming strategy

## Abstract

Oncolytic virotherapy has emerged as a promising treatment for human cancers owing to an ability to elicit curative effects *via* systemic administration. Tumor cells often create an unfavorable immunosuppressive microenvironment that degrade viral structures and impede viral replication; however, recent studies have established that viruses altered *via* genetic modifications can serve as effective oncolytic agents to combat hostile tumor environments. Specifically, oncolytic vaccinia virus (OVV) has gained popularity owing to its safety, potential for systemic delivery, and large gene insertion capacity. This review highlights current research on the use of engineered mutated viruses and gene-armed OVVs to reverse the tumor microenvironment and enhance antitumor activity *in vitro* and *in vivo*, and provides an overview of ongoing clinical trials and combination therapies. In addition, we discuss the potential benefits and drawbacks of OVV as a cancer therapy, and explore different perspectives in this field.

## Introduction

Over the last decade research attention has shifted from cancer cells to the tumor microenvironment (TME) given that approximately 95% of cancer cases stem from the environment and lifestyle^1^. Cancer cells exist and continue to thrive within the TME, which is comprised of cancer and non-cancerous host cells (such as immune cells), microvessels, and various cytokines and chemokines. Interactions between tumor cells and the TME have crucial roles in tumor development, progression, metastasis, and therapeutic responses^1,2^. The success of emerging immunotherapies indicates that the most promising approach is harnessing the immune system to fight against cancer^3^. There are several types of immunotherapy, including immune checkpoint inhibitors (ICIs), chimeric antigen receptor T (CAR-T) cell therapy, bacterial therapy, and oncolytic virotherapy. ICIs include antibodies, ligands, and Fc-fused proteins that interact with innate and adaptive immune receptors, such as PD-1/PD-L1, CTLA-4, TIM-3, LAG-3, TIGIT, CD47, and SIRPα. ICIs have shown promise in the treatment of several types of cancer; however, monotherapy is unlikely to yield long-term benefits for most patients due to severe immune-related adverse events^4–6^. CAR-T cell therapy involves arming modified T cells that navigate to target cancer cells *via* CD19 and BCMA. This method is particularly effective for the treatment of haematologic malignancies; however, the effectiveness against solid tumors has been limited. Therefore, new therapeutic strategies are needed to overcome this resistance.

Oncolytic viruses (OVs) are an attractive therapeutic strategy. In general, oncolytic virotherapy benefits from tumor cell selective replication and oncolysis, which lead to a chain reaction of immune activation. Serving as a therapeutic vector that delivers exogenous therapeutic genes to amplify anti-tumor responses and restore anti-tumor immunity, which reprograms the TME by various mechanisms is another advantage. This armed OV strategy has been successfully tested in preclinical studies and clinical trials. For example, OV can be armed with cytokines, chemokines, monoclonal antibodies, bispecific antibodies, ligands, enzymes, and suicide genes. These therapeutic genes are mainly involved in immune activation, immune stimulation, and cell metabolism. To date, only the herpes simplex virus type 1 OV, talimogene laherparepvec (T-VEC), has been approved by the U.S. Food & Drug Administration^7,8^. Several virus species, including adenovirus, herpes simplex virus, and vaccinia virus, have been designed for immunotherapy^9^; however, safety concerns regarding OVs that can infect healthy cells or cause unintended side effects have limited clinical application. Because of the different structures and physiologic features, OVs have dissimilar abilities to dissolve tumors, induce immune responses, and capabilities to insert foreign genes^10^. Compared to RNA viruses, DNA viruses, such as vaccinia virus (VV), have larger and more complete genomes, making DNA viruses easier to manipulate and pack larger transgenes^11^. **Table 1** summarizes the strengths and shortcomings of VV and other OVs. VV has been utilized as a smallpox vaccine by the World Health Organization since 1976, thus there is significant experience and in-depth clinical knowledge of the vaccine^12^. As of 2023, there have been approximately 30 reported clinical trials involving the oncolytic vaccinia virus (OVV) for the treatment of melanoma, and ovarian, colorectal, and hepatocellular carcinomas, with > 1500 cancer patients treated. In this study we provide an overview of different OVV strains and mutations in multiple transgenes that have reversed the immunosuppressive TME in a series of preclinical studies and related clinical trials. In addition, reciprocal inhibition between cancer cells and the OVV within the TME is discussed.

**Table 1:** Characteristics of VV and other OVs

Virus	VV	Adenovirus	Herpes simplex virus	Coxsackievirus
Genome	dsDNA(∼200 kb)	dsDNA(30–40 kb)	dsDNA(∼152 kb)	(+)ssRNA(∼7.4 kb)
Genome capacity	25–40 kb	7–8 kb	30–40 kb	300 bases
Cell entry mechanism	Membrane penetration and fusion	Endocytosis	Endocytosis; penetration	Micropinocytosis
Replication site	Cytoplasm	Nucleus	Nucleus	Cytoplasm
Risk of integration	No	More risk	More risk	No
Immunogenicity	High	Low	Low	Low

## Improvement of tumor cell immunogenicity within the TME

### OVV directly increases tumor cell immunogenicity

Generating an effective immune response against tumors is challenging because of the limited number of tumor antigens and low immunogenicity, especially among ‘cold’ tumors. These features present significant barriers to successful cancer immunotherapy. OVVs selectively infect tumor cells and replicate continuously, thus causing oncolysis^13^. The viral progeny released from disrupted tumor cells infect peripheral tumor cells, leading to a positive chain-like infection that produces a long-lasting anti-tumor effect^14^. When OVVs infect tumor cells, tumor-associated molecular patterns are exposed^15,16^, such as tumor-associated antigens (TAAs), danger signals (DAMPs), PAMPs, and cytokines, which trigger local immune responses. Additionally, tumor-associated molecular patterns stimulate innate and adaptive immunity at distal tumor sites that are not directly injected by the virus^17^.

Several mechanisms promote cancer cell death in the TME. OVV-white-spotted char lectin (WCL) promotes tumor cell apoptosis through the activation of caspase-3 and cleaved caspase-9, increases the level of interferon expression, and inhibits tumor growth *in vivo*^18^. Furthermore, OVVs have been modified to include genes that promote apoptosis, such as second mitochondria-derived activator of caspase (SMAC) and tumor necrosis factor-related apoptosis-inducing ligand (TRAIL). These modifications increase cytotoxicity and induce apoptosis in pancreatic cancer tissues, as well as other cancer models^19–21^. Recently, we developed an OVV expressing the autophagic gene Beclin-1 (named OVV-BECN1). After OVV-BECN1 infection, translated Beclin-1 activates downstream signalling molecules, resulting in autophagosome formation and the induction of autophagic cell death. It has a favourable therapeutic effect in leukemia and multiple myeloma models^22^. Additionally, an OVV expressing the *FCU-1* suicide gene, TG6002, catalyses the conversion of 5-fluorocytosine (5-FC) to 5-fluorouracil and has shown significant antitumor activity, especially when used in combination with external 5-FC administration^23^.

In the immunosuppressive TME, the upregulated expression levels of suppressive cytokines impedes antitumor immunity and impairs the efficacy of ICIs in clinical settings^24^. To this end, OVVs expressing ICIs may increase tumor immunogenicity and reverse immunosuppressive signals within the TME. For instance, an engineered OVV carrying a gene encoding a full anti-TIGIT antibody was developed to suppress immune responses activated by single-agent OV treatment. Using mouse models, vaccinia virus-α-TIGHT demonstrated efficacious antitumor immunity, long-term efficacy, and the establishment of immunological memory involving CD8+ T cells and natural killer (NK) cells^25^. Similarly, our research group conducted a preliminary study showing that an engineered OVV expressing an anti-CD47 nanobody improves efficacy against lymphoma by promoting macrophage phagocytosis of CD47+ tumors and NK-cell-mediated antibody-dependent cellular cytotoxicity. Another group obtained similar results using herpes simplex virus^26^. These studies underscore the potential of OVV as a promising vector to turn ‘cold’ tumors into ‘hot’ tumors by promoting the infiltration of immune cells^27^.

### OVVs indirectly increase tumor cell immunogenicity

Despite the infiltration of immune cells and pro-inflammatory cytokines into immune-desert cold tumors, the tumors remain immune-resistant^28^. The effect of cytokines on immune cells depends on the properties, concentration, and environment; cytokines may either activate or inhibit immune cells^29^. OVVs bearing cytokines, such as GM-CSF, IL-2, IL-6, IL-12, IL-15, IL-23, and IL-24, have demonstrated exceptional anti-cancer effects and remarkable safety in clinical and preclinical evaluations. EphA2-TEA-OVV, which expresses a bispecific T cell engager targeting CD3, EphA2, or EpCAM, exhibits notable anti-tumor activity and bystander killing of non-infected tumor cells^30,31^. Our team developed an OVV encoding a bispecific T cell engager (OVV-CD19BiTE) that activates and induces memory T cell differentiation, which led to an effective treatment of B-cell lymphoma^32^.

The combination of OVs and hormonal factors is a promising approach for cancer therapy. Prostaglandin E2 (PGE2), a vital homeostatic hormone, is a key mediator in cancer immunity. The COX-2/PGE2 axis contributes to the development of therapeutic resistance and inhibits immune activation in the TME. Targeting PGE2 using OVV-engineered hydroxy prostaglandin dehydrogenase 15 (15-HPGD) or the COX-2 inhibitor, celecoxib, reverses the immunosuppressive state and reduces the number of immunosuppressive cells in the TME, which leads to improved therapeutic outcomes^33^. Another recent study revealed that an OVV engineered to express leptin, which functions as a metabolic modulator, alters the status of T cells in the TME. Environmental leptin enhances mitochondrial function and the oxidative phosphorylation of tumor-infiltrating T cells, thus providing metabolic support for these immune cells^34^. This study on the combination of an OVV and hormonal factors shed new light on OV therapy and suggested that metabolic reprogramming, rather than solely the influx of specific immune cells into the TME, may play a superior role in inhibiting tumor growth.

## Enhanced OVV durability and selectivity to overcome the immunosuppressive TME

### Characteristics of VV

**Figure 1:**
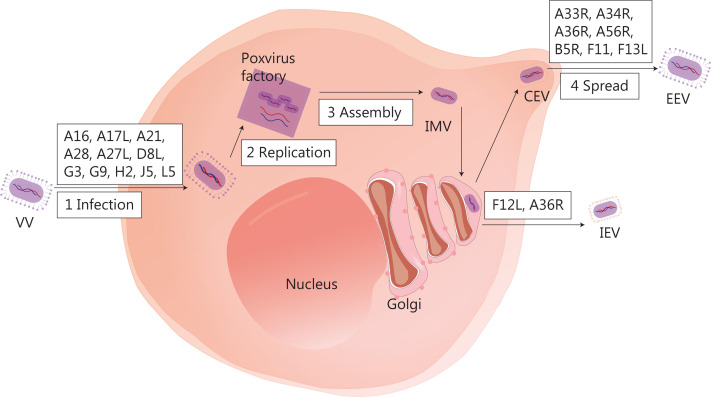
The life cycle of VV and major viral proteins involved in virus formation and transmission. The virus forms a fusion protein complex that consists of eight viral proteins (A16, A21, A28, G3, G9, H2, J5, and L5), then enters the cell interior. IMV, as an infectious form, has A17L, A27L, and D8L to help adhere to the surface of the cell membrane. VV replication and progeny assembly occur in the “poxvirus factory” of the cytoplasm of infected cells. IMV is transported to the extracellular space through microtubules, while fusing with the cell membrane to form CEV. CEV encodes genes (A33R, A34R, A36R, A56R, B5R, and F13L), thus forming EEV for intercellular transmission and distant metastasis. IMV can also be encapsulated by the Golgi complex to form IEV, which is then transported to the periphery of cells mediated by F12L and A36R. IMV, intracellular mature virus; CEV, cell-associated enveloped virus; EEV, extracellular enveloped virus; IEV, intracellular enveloped virus.

Several biological characteristics of VV make it an excellent viral platform for cancer immunotherapy. First, VV is a large enveloped virus with a linear double-stranded 190-kb DNA genome that encodes approximately 250 genes, but can accommodate up to 50 kb of transgenes^35^. Second, VV requires host cells for replication, and the replication cycle is dependent on a high-fidelity DNA polymerase, which ensures the integrity of the viral genome^36^. Third, the virus replicates exclusively in the cytoplasm^37^, thus minimising the risk of viral DNA integration into the host genome and making it an excellent OV candidate. Fourth, compared to other OVs, the VV has some advantages, such as rapid replication (progeny viruses can be produced in 6 h)^38^, broad tumor tropism, and the ability to replicate without limitations under hypoxic conditions. Fifth, VV infection prompts immune responses without causing significant disease in healthy individuals. The vast amount of data on the long-term use of VV vaccines provides a strong safety foundation for clinical application. Antiviral drugs can be used to manage the potential adverse effects^39,40^. Sixth, even if patients receive a VV or produce neutralising antibodies, the virus can still effectively infect the tumor *via* intravenous injection. Finally, the virus has three transmission mechanisms: cell-to-cell spread^41^, extracellular enveloped virus release^42^, and repulsion of superinfecting virions^43^. Of note, the details of these complex mechanisms are not well understood. **Figure 1** depicts the VV life cycle, with a diagram of infected cells and the important viral proteins involved in virus formation and transmission.

### Strategy for the modification of OVVs

An unmodified VV has the ability to kill tumor cells, but the clinical application is limited due to its side effects, such as hepatitis and fatal neuroencephalitis, as reported in historical clinical data from the 1980s^44^. To improve clinical efficacy, modifications have been made to the virus with the goal of better tumor cell selectivity, stronger target gene expression, higher therapeutic effects, lower toxicity, and less immunogenicity. As shown in **Table 2**, vaccinia vectors used for oncolytic utilities mainly include the Wyeth^45^, Western reserve (WR)^46^, Lister^47^, Copenhagen^23^, New York City Board of Health (NYCBH)^48^, and Tiantan (TTV) strains^49^. Thymidine kinase (TK) is the most commonly deleted gene. TK is one of the key enzymes for the synthesis of VV DNA. Deletion of the *J2R* gene (encoding TK) makes VV a tumor-selective cloning and expression vector, and VV has been further confirmed to be associated with decreased virulence of recombinant VVs, such as Pexa-Vec. VV is an extracellular enveloped virus (EEV) that contains a host-derived outer membrane and B5R protein, which can be recognised by the complement system and results in EEV neutralisation^50–52^. Partial deletion of short consensus repeats in the *B5R* gene increases neutralisation escape, without affecting the oncolytic potency of the VV, making VV resistant to immune clearance and improving therapeutic outcomes^49^.

**Table 2:** Oncolytic vaccinia virus strains and mutations studied for cancer treatment

Strain	Modification	Representative viruses	Insertions (Ref)
Wyeth	*J2R*-	Pexa-vec	GM-CSF^45^
WR	*J2R-*, *VGF*-	vvDD	GFP^46^
Lister	*J2R*-, *F14.5L*-, *A56R*-	GL-ONC1	Renilla luciferase-GFP fusion protein, β-galactosidase, β-glucuronidase^74^
	*VGF*-, *O1L*-	ASP9801	IL-7/IL12^57^
Tian Tan Guang 9	*J2R*-	VG9	GM-CSF; IL-24^95^
Copenhagen	*J2R*-, *I4L*-	TG-6002	FCU-1^23^
New York City Board of Health	5p, 3p, and B14R	vaccinia virus-aCEA TCE	aCEA T cell engagers^29^

Given the complex interactions between the immune system and OVV, a single disruption of *J2R* may not be sufficient. Thus, double-, triple-, and quadruple-deletion mutant VVs have been generated. *I4L* encodes a large subunit of ribonucleotide reductase (RR). The *J2R-* and *I4L*-mutant virus, TG6002, exhibits uninfluenced tumor-selective replication, tumor cell killing, and highly-attenuated virulence in healthy cells compared to the single TK-deleted version^23^. Similarly, the deletion of *F4L*, the gene encoding the small subunits of RR,^53^ and *J2R* yield similar results^54^. Pelin et al.^55^ generated an OVV by deleting *J2R* and the anti-apoptotic viral gene, *F1L*. This double mutation not only increased the safety of the Copenhagen-strain-derived OVV, but also improved the ability of the OVV double mutation to induce cancer cell death. Another *J2R*-based double-deleted OVV target, B18R, is a type I interferon inhibitor. Additional deletion of B18R rendered the virus carrying interferon-beta with superior tumor selectivity and systemic intravenous efficacy in animal models^56^. The double-mutated vaccinia virus, vvDD-GFP, which has a deletion of *J2R* and *VGF* (an epidermal growth factor homologue encoded by the *C11R* gene that promotes infected cell motility and spread of the viral infection), shows no toxicity in healthy cells, but significant tumor regression is observed at high doses in nude mice^46^. Triple mutation of *VGF*, *O1L* (continuously activates extracellular signal-regulated kinase 1/2 and promotes viral virulence), and *B5R*^57^, as well as J2R, F14.5L, and A56R (encoding hemagglutinin, mediates viral attachment to host cells and inhibits the fusion of infected cells)^47^ give similar results in selective replication and reduced toxicity. Viruses with these mutations are excellent candidates for promoting therapeutic effects owing to superior safety indices. It is interesting to note that OVVs with the quadruple mutation of *J2R*, *A48R* (encoding thymidylate kinase, an enzyme that participates in nucleotide metabolism), *B18R*, and *C11R* maintain tumor selectivity. In a melanoma model, strong viral attenuation, reduced virus dissemination, and effective anti-tumor activity were observed as expected^58^.

All VVs with multiple mutations enhance the selectivity of tumor cells, mitigate virulence, and manifest either unaltered or amplified tumor control. Significant functional improvements owing to multiple deletions have become a popular strategy for OVV development. Recently, quintuple deletions (*C7L-K2L*, *E3L*, *A35R*, *B13R*, and *A66R*) in the VV Tian Tan strain enabled the generation of a more powerful OVV to treat cancer^59^. In addition to improving the selectivity of the OVV, increasing the viral replication ability through gene modification is also important. Using small interfering RNA screening technology, Liu et al.^60^ identified a relationship between the essential necroptosis kinase receptor interacting protein kinase 3 (RIPK3) and the viral inducer of RIPK3 degradation (vIRD). vIRD promotes the ubiquitination and proteasome-mediated degradation of RIPK3, thereby promoting viral replication. This phenomenon has also been observed in a mouse model. It is expected that more efficient OVVs will be designed for cancer therapy in future studies.

### Accurate targeting of the virus

Direct injection into the tumor site has a limited systemic impact, whereas intravenous and intraperitoneal infusions have broader effects, but face difficulties in effectively targeting the tumor. The two main factors that promote virus-specific replication in cancer cells are EGFR/Ras pathway activity and cellular TK levels^44,61,62^. Some VVs lacking TK, such as JX-594^63–67^ armed with GM-CSF, GL-ONC1^68,69^, vvDD^70,71^, TG6002^23^, and T601, have been developed, whereas other VVs that are TK-positive have achieved good clinical results^72^. OV vectors can be genetically modified to target tumor-associated surface markers, such as MUC1^73^, to improve efficacy. OVVs have also been used to deliver surface antigens to CAR-T cells^74^ that recognise target cells based on antigen density, as demonstrated by the successful delivery of CD19 to B16 melanoma cells using a TK-disrupted VV^75^. The results were promising, showing a significant improvement in the tumor-killing ability of CAR-T cells and an increase in median survival. Furthermore, OV vectors can be engineered to produce cytokines or chemokines that enhance CAR-T cell function and anti-tumor efficacy, as demonstrated by an engineered VV that produces CXCL11, resulting in increased CXCL11 protein levels and antigen-specific T cell numbers in tumors^76^. These findings suggest that OV vectors hold promise for overcoming the current challenges facing CAR-T cell therapy in solid tumors, and using CAR-T cell guidance enhances VV targeting of tumor cells. Future clinical trials are necessary to validate these findings and to advance the use of OVs in cancer therapy.

## Clinical trials of OVVs

In 1995, a phase 3 randomized, double-blind, multi-centre trial of vaccinia melanoma oncolysate (VMO) in patients with stage II melanoma was conducted. There was no difference in the disease-free interval or overall survival between the active specific immunotherapy with VMO and placebo groups^77^. Despite this setback, further research on VVs for cancer immunotherapy has continued, with modifications aimed at improving the effectiveness of treatment based on the physiologic characteristics of the virus and previous experience.

Given the role of cytokines and chemokines in the inflammatory environment of the TME, an OVV (JX-594) was modified to include GM-CSF. In 2013, a randomised phase II trial involving JX-594 in liver cancer showed that the subject survival duration was significantly dependent on the dosage (median survival of 14.1 months *vs.* 7 months on high and low doses, respectively; hazard ratio = 0.39; *P* = 0.020)^67^. Recently, a phase I/II study of JX-594 was conducted in combination with an ICI for refractory metastatic colorectal cancer (mCRC). In this study there was no significant difference in the median progression-free survival (PFS) between the PexaVec/durvalumab/tremelimumab cohorts and the Pexa Vec/durvalumab cohorts (2.3 months *vs.* 2.1 months; *P* = 0.57)^65^, but the number of Ki67+CD8+ T cells increased in peripheral blood mononuclear cells. Further studies are required to determine the potential clinical activity of the combination of VV and ICIs.

To improve immunogenicity, VVs have been armed with genes encoding specific TAAs and co-stimulatory molecules. In 1996, a recombinant vaccinia virus (TA-HPV) was engineered to encode human papillomavirus (HPV) types 16, 18, E6, and E7 proteins, and used as immunotherapy for cervical cancer. Although the clinical effectiveness was limited by the sample size, 2 patients remained clinically well at 15 and 21 months post-vaccination^78^. Later, a phase I trial for metastatic melanoma patients was conducted using a recombinant vaccinia virus expressing B7.1 (rV-B7.1), which showed that rV-B7.1 induced objective tumor regression, anti-VV antibody responses, and T cell responses against defined melanoma antigens^79^. Kantoff et al.^80^ conducted a phase II randomised controlled trial of PROSTVAC-VF in prostate cancer in 2010 that was comprised of two recombinant viral vectors, each encoding a transgene for PSA and three immune co-stimulatory molecules (B7.1, ICAM-1, and LFA-3). The PROSTVAC-VF group had better overall survival 3 years post-study, with 25 (30%) of 82 treated patients alive versus 7 (17%) of 40 control patients alive, and longer median survival by 8.5 months (25.1 months for treated patients *vs.* 16.6 months for controls)^81^.

Based on recent preclinical research and clinical trials (**Table 3**), OVVs were shown to have great promise as a platform for various approaches to eliminate cancers. Genetic modification of these viruses can significantly enhance their ability to control tumors, making it a promising method for improving therapeutic efficacy and overcoming some of the clinical limitations.

**Table 3:** Key clinical trials of OVVs from ClinicalTrials.gov

Name	Genetic modifications	Enrolment	Interventions	Indication	Clinical responses	Status	NCT number
**Pexa-Vec (JX-594)**	Wyeth strain (ΔTK) Transgenic expression of GM-CSF and β-galactosidase	34	+Drug: Durvalumab (anti-PD1); Tremelimumab (anti-CTLA-4)	Colorectal cancer	Median PFS 2.3 months (PexaVec/durvalumab/tremelimumab cohorts) *vs.* 2.1 months (the PexaVec/durvalumab cohorts)	Phase I; Phase II	NCT03206073^65^
		10	Monotherapy	Melanoma	DOR and PFS were not assessable since most patients went off study within 6 weeks	Completed Phase I; Phase II	NCT00429312^109^
		15	Monotherapy	Colorectal carcinoma	PFS and OS of all eligible patients were 61 days and 10.3 months	Completed Phase II	NCT01469611^63^
		23	+Drug: Sorafenib	Carcinoma, hepatocellular	FGF-2 stimulated JX-594 activation in endothelial cells; JX-594 was able to specifically target and infect TECs	Completed Phase II	NCT01171651^110^
		14	Monotherapy	Neoplasms, liver	Median survival for all 14 patients was 9 months	Completed Phase I	NCT00629759^45^
		6	Monotherapy	Neuroblastoma	4 of 6 patients had a SD and 2 had PD in the injected target lesion	Completed Phase I	NCT01169584^111^
		30	Monotherapy	Carcinoma, hepatocellular	Median OS 14.1 months (high dose) *vs*. 6.7 months (low dose)	Completed Phase II	NCT00554372^67^
		129	Monotherapy	Carcinoma, hepatocellular	Median OS 4.2 months (Pexa-Vec+BSC) *vs*. 4.4 months (BSC)	Completed Phase II	NCT01387555^112^
		23	Monotherapy	Melanoma	Dose-related antitumor activity was correlated with delivery and replication of JX-594	Completed Phase I; Phase II	NCT00625456^40^
**GL-ONC1 (GLV-1h68)**	Lister strain (ΔF14.5L, ΔA56R and ΔTK) Transgenic expression of Luc-GFP, β-glucuronidase	64	+Drug: Chemotherapy or bevacizumab (anti-EGFR)	Ovarian cancer	Median OS 18.5 months (platinum-resistant group) *vs.* 14.7 months (platinum-refractory group)	Completed Phase I; Phase II	NCT02759588^113^
		19	Monotherapy	Cancer of the head and neck	With median follow-up of 30 months, 1-year (2-year) PFS and OS were 74.4% (64.1%) and 84.6% (69.2%), respectively	Completed Phase I	NCT01584284^69^
		9	Monotherapy	Peritoneal carcinomatosis	GL-ONC1 was well tolerated when administered into the peritoneal cavity of patients with advanced stage peritoneal carcinomatosis	Completed Phase I; Phase II	NCT01443260^68^
**TroVax**	Modified vaccinia virus Ankara Transgenic expression of tumor antigen 5T4	733	+Drug: Sunitinib	Renal cell cancer	No significant difference in OS was evident in the two treatment arms; The magnitude of the 5T4-specific antibody response induced by vaccination with MVA-5T4 was associated with enhanced patient survival	Completed Phase II/III	NCT00397345^114^
**TG4010**	Modified vaccinia virus Ankara Transgenic expression of MUC1 and IL-2	222	+Drug: First-line chemotherapy	Non-small-cell lung carcinoma	Median PFS 5.9 months (TG4010 group) *vs.* 5.1 months (the placebo group)	Terminated Phase II/III	NCT01383148^115^
**PROSTVAC**	Wyeth strain (ΔTK) Transgenic expression of B7.1, ICAM-1, and LFA-3	120	Monotherapy	Prostate cancer	PROSTVAC-VF immunotherapy was well-tolerated and associated with a 44% reduction in the death rate and an 8.5-month improvement in the median OS	Completed Phase II	NCT00078585^80^
**PANVAC**	Wyeth strain (ΔTK) Transgenic expression of CEA, MUC-1, B7.1, ICAM-1, and LFA3	48	+Drug: Docetaxel	Breast cancer	Median PFS 7.9 months (combination group) *vs.* 3.9 months (control group)	Completed Phase II	NCT00179309^116^
**MVA-5T4**	Modified vaccinia virus Ankara Transgenic expression of tumor antigen 5T4	25	Monotherapy	Renal cell cancer	Vaccination with MVA-5T4 did not improve ORR of IL-2 therapy, but did result in SD associated with an increase in the ratio of 5T4-specific effector-to-regulatory T cells in selected patients.	Completed Phase II	ISRCTN 83977250^117^
**rV-B7.1**	Wyeth strain (ΔTK) Transgenic expression of B7.1	12	Monotherapy	Melanoma	Melanoma patients injected with rV-B7.1 develop anti-vaccinia virus antibody responses and T cell responses against defined melanoma antigens	Completed Phase I	NCT00004148^79^
**IN rVV**	Copenhagen strain (ΔTK and ΔI4L)	15	Monotherapy	Melanoma	Of 10 remaining patients 7 showed evidence of induction of CTLs directed against at least one epitope	Completed Phase I/II	Not found^117^

PFS, progression-free survival; DOR, durability of responses; TECs, tumor-associated endothelial cells; SD, stable disease; PD, progressive disease; BSC, basic support care; ORR, objective response rates; CTLs, cytotoxic T lymphocytes. Some clinical trials without publicly available results have not been included in the table.

### Production of neutralizing antibodies

Concerns about the efficacy of the smallpox vaccine in older patients vaccinated at a young age remain a general issue for VV. The antiviral response, particularly the production of neutralising antibodies, may persist in the TME. In a phase I study, it was shown that individuals > 45 years of age displayed minimal anti-VV antibody levels before treatment with VV. Although neutralising antibodies were shown to increase rapidly within 3–6 weeks of treatment, OV proliferation, replication, and tumoricidal properties were not affected^40,82^. Research from decades ago showed that the unique biology of VV allows for the production of ‘invisible’ particles (EEVs) that can safely travel in the blood in the presence of neutralising antibodies and complement. Therefore, repeated intravenous injections may theoretically serve as an effective therapeutic tool for enhancing immunity^83,84^.

### Drug dosage

An appropriate dosage, based on efficacy and safety, is critical for achieving excellent curative effects. The hostile TME, which is characterised by hypoxia and antiviral reactions, makes survival at low doses challenging^9,37,85^. Elevating the concentration of specific cytokines around tumors can indirectly prolong viral survival in tumors treated with low doses^86,87^. Preclinical studies have demonstrated that increased concentrations of IL-10 and IL-23 in the TME can promote viral replication, prolong viral survival, and inhibit immune responses. In a phase I trial of intra-tumoral injection into solid tumors, JX-594 was well-tolerated, endogenous cytokine levels increased in a dose-dependent manner^40^, and challenges associated with high-dose administration were well-documented. An open-label, three-step, dose-escalating, single-centre, phase Ib study involving Olvi-Vec, a modified vaccine virus, was conducted. To assess safety by analysing adverse events, Olvi-Vec was divided into three dose groups, and each group was administered intraperitoneally^82^. Surprisingly, no differences in toxicity were observed between the three dose groups. Of the 11 subjects in the study, stable disease was observed in 7 individuals, with an overall response rate of 9% (1/11) and a median PFS of 15.7 weeks (95% confidence interval: 5.7–34.5). In another phase II clinical trial involving JX-594, some patients in the high-dose group had symptoms of lymphopenia and were evaluated for serum transaminase concentrations (2-week duration); however, despite these adverse effects, the high-dose treatment group did not show reduced efficacy, exhibiting longer-term survival benefits than the low-dose cohort (median survival of 14.1 months *vs.* 6.7 months, hazard ratio = 0.39; *P* = 0.020)^67^.

As many genes encoded by the VV genome have unknown functions, unpredictable challenges remain. In addition, although VV prefers cells with rapid cell cycle progression, similar to cancer cells, VV can also infect various cell types, including somatic cells^88^. Therefore, modifying viral strains with targeted mutations in viral genes related to nucleotide metabolism, apoptosis, inflammation, chemokines, and interferon signalling can improve tumor selectivity, safety, viral replication, and the ability to modulate immune responses, while maintaining viral replication and oncolytic capacity^6,55,89–91^.

## Discussion

Oncolytic virotherapy holds promise as a cancer treatment, but often exhibits limited therapeutic efficacy when used alone, which is a persistent concern across the field of cancer therapy. To address this issue, innovative modifications and combination therapies are required to improve the effectiveness of OVs. In this review we discussed the advantages and disadvantages of OVVs and highlighted several critical considerations for future development.

The safety of the VV for human use has been established based on its previous application as a vaccine against smallpox and its ability to replicate exclusively in the cytoplasm, precluding its integration into the host genome. Another significant advantage of VV is its capacity for intravenous administration, which sets it apart from many other OVs that are limited to intra-tumoral injections. This approach is impractical for patients with superficial tumors and multifocal metastases. While the intravenous infusion of VV may be susceptible to host antiviral immune response, a recent study using transient pharmacologic inhibition of leukocyte-enriched phosphoinositide 3-kinase δ (PI3Kδ) demonstrated successful improvement of VV delivery to tumors in mouse models^92^.

Despite the widespread use of OVVs in preclinical and clinical studies, several obstacles remain. The TME is the microecology upon which tumor cells depend for survival and where OVs exhibit anti-tumor effects, making the TME crucial for both tumors and viruses. Owing to its significant immunogenicity, the VV is quickly eliminated by the host’s immune system, which restricts its oncolytic potential and replication. The modification of viral vectors is required to reduce immunogenicity and improve immune evasion. Additionally, the large size of the VV necessitates stringent sterile conditions for its production and preparation. Therefore, oncolytic virotherapy should be tailored to specific cancer types by developing biomarker panels to determine the sensitivity of tumors to this therapeutic approach.

**Figure 2:**
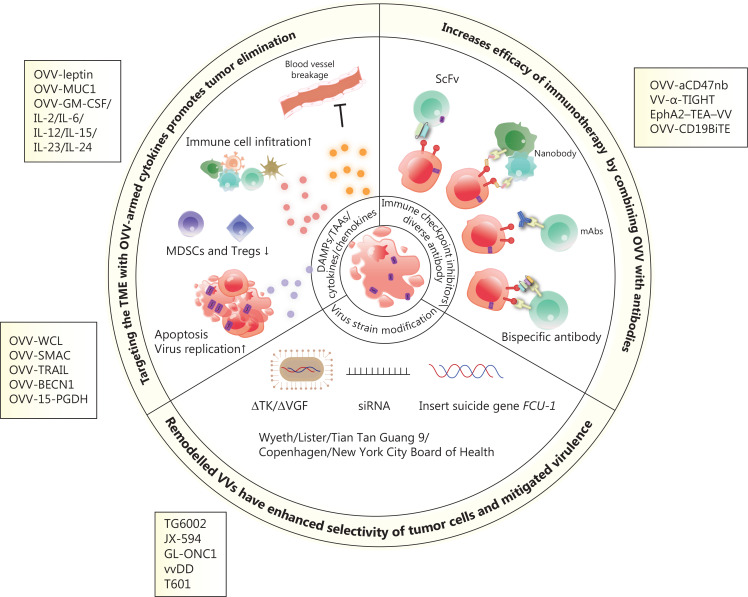
Scheme of engineered oncolytic vaccinia virus (OVV) and mechanisms of enhanced anti-tumor activity. OVVs armed with cytokines or tumor suppressor genes enhance immune cell infiltration, damage the vascular bed, inhibit suppressive immune cells (such as MDSCs and Tregs), and induce cell apoptosis and autophagy, thus resulting in the release of tumor-associated antigens. OVVs express immunotherapeutic genes, including those encoding immune checkpoint inhibitors, antibodies, and bispecific antibodies, which exert potent and specific cytotoxicity in a variety of tumor models by enhancing immunotherapeutic effects. Modified OVVs with a thymidine kinase (TK) deletion, the insertion of a suicide gene, and expression of siRNA have increased oncolytic properties and safety.

**Figure 3:**
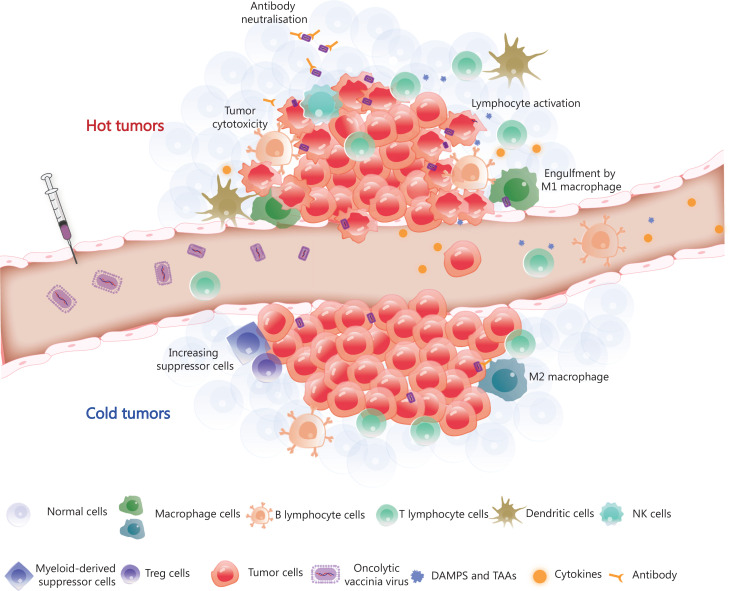
Tumor microenvironment remodelling induced by OVV. The OVV can be administered intravenously, after which it selectively infects and replicates in tumor cells. OVV is then released within the tumor microenvironment (TME), resulting in a change in the TME from the original ‘cold’ state (immunosuppression) to a ‘hot’ state (immune activation) due to the infiltration of immune cells. Macrophages and dendritic cells engulf OVV-infected tumor cells and present antigens to lymphocytes. CD8+ T lymphocytes work in coordination with immune checkpoint inhibitors or immunotherapeutic antibodies released by OVV-infected tumor cells and eliminate the tumor cells. The armed antibody with an Fc fragment results in the activation of natural killer (NK) cells, thereby stimulating the antibody-dependent cellular cytotoxicity of NK cells. DAMPs, danger-associated molecular patterns; TAAs, tumor-associated antigens; Treg cells, regulatory T cells.

The VV genome encodes approximately 250 genes involved in virulence and suppression of the immune system. Removing specific genes and inserting immunostimulatory genes or other genes into the viral genome are fundamental and promising methods for constructing recombinant viruses. The sizeable viral genome of the VV allows for the introduction of up to 50 kb of foreign genes, making VV a productive therapeutic tool for multiple gene delivery, including genes encoding antibodies^31,93^, cytokines^57,94–96^, chemokines^97,98^ and ligands^99^ (**Figure 2**). Oncolytic virotherapy has the potential to be an attractive combination partner with other immunotherapies based on the mechanisms of tumor resistance to immune-mediated clearance. This feature provides an intense synergistic effect that maximises the benefits of combination therapy, as reported in previous studies. The OVV represents an ideal element for combination therapy because of its good safety profile and multiple anti-tumor mechanisms. Viral infection and capacity to lyse tumors converts ‘cold’ tumors into ‘hot’ tumors and enhances the infiltration and recruitment of immune cells into the TME (**Figure 3**).

An OVV combined with immune checkpoint inhibition exhibits a potent synergistic effect. Moreover, OVVs armed with monoclonal antibodies^93^, bispecific antibodies^100^, and functional ligand-specific^99^ have also been demonstrated to have anti-tumor effects in a series of preclinical studies. Deliberate therapeutic regimens are necessary, however, for combined therapies because ICIs can hinder VV replication^101^. It is crucial to achieve both effects for maximum results, while avoiding the negative effect of the armed gene in the OVV. Simultaneous treatment with an OVV and ICIs results in more effective therapeutic outcomes than single treatments^98^; however, integrating genes expressing ICIs into the viral genome may be an ideal approach for combination therapy with OVVs^102–108^.

Overall, the effectiveness of oncolytic VV-based therapies has been demonstrated in a wide range of cancer types in many studies. Recently, various strategies that can be synergistically implemented for maximum efficacy have been developed. Among them, combination therapies with immunotherapy could be a very promising approach to improve therapeutic outcomes.
